# Analysis of urinary potassium isotopes and association with pancreatic health: healthy, diabetic and cancerous states

**DOI:** 10.3389/fendo.2024.1332895

**Published:** 2024-04-02

**Authors:** Kathrin Schilling, Heng Chen, Ronald A. Glabonjat, Silvana Debernardi, Oleg Blyuss, Ana Navas-Acien, Alex N. Halliday, Tatjana Crnogorac-Jurcevic

**Affiliations:** ^1^ Department of Environmental Health Sciences, Columbia University Mailman School of Public Health, New York, NY, United States; ^2^ Lamont‐Doherty Earth Observatory, Columbia University, Palisades, NY, United States; ^3^ Centre for Cancer Biomarkers and Biotherapeutics, Barts Cancer Institute, Queen Mary University of London, London, United Kingdom; ^4^ Wolfson’s Institute for Cancer Prevention, Queen Mary University of London, London, United Kingdom

**Keywords:** potassium isotopes, urine, pancreas, cancer, diabetes, dysregulation

## Abstract

**Background:**

More than 700 million people worldwide suffer from diseases of the pancreas, such as diabetes, pancreatitis and pancreatic cancer. Often dysregulation of potassium (K^+^) channels, co-transporters and pumps can promote development and progression of many types of these diseases. The role of K^+^ transport system in pancreatic cell homeostasis and disease development remains largely unexplored. Potassium isotope analysis (δ^41^K), however, might have the potential to detect minute changes in metabolic processes relevant for pancreatic diseases.

**Methods:**

We assessed urinary K isotope composition in a case-control study by measuring K concentrations and δ^41^K in spot urines collected from patients diagnosed with pancreatic cancer (n=18), other pancreas-related diseases (n=14) and compared those data to healthy controls (n=16).

**Results:**

Our results show that urinary K^+^ levels for patients with diseased pancreas (benign and pancreatic cancer) are significantly lower than the healthy controls. For δ^41^K, the values tend to be higher for individuals with pancreatic cancer (mean δ^41^K = -0.58 ± 0.33‰) than for healthy individuals (mean δ^41^K = -0.78 ± 0.19‰) but the difference is not significant (p=0.08). For diabetics, urinary K^+^ levels are significantly lower (p=0.03) and δ^41^K is significantly higher (p=0.009) than for the healthy controls. These results suggest that urinary K^+^ levels and K isotopes can help identify K disturbances related to diabetes, an associated factors of all-cause mortality for diabetics.

**Conclusion:**

Although the K isotope results should be considered exploratory and hypothesis-generating and future studies should focus on larger sample size and δ^41^K analysis of other K-disrupting diseases (e.g., chronic kidney disease), our data hold great promise for K isotopes as disease marker.

## 1 Introduction

Use of stable and isotopes in modern medicine has demonstrated their potential to answer a wide array of research questions, relevant for diagnosis, prevention, and management of diseases (1–6). Disease development can change metabolic processes which can be revealed in stable isotope ratios (heavy *vs* light isotopes) in tissue, urine and blood (1–6). Typical changes in natural isotopic composition in the body are one order of magnitude greater than their measurement uncertainties (≈0.02‰), which allows for precise measurements and comparisons.

Potassium (K^+^) is essential for all life. It has three naturally occurring isotopes: ^39^K (93.258%), ^40^K (0.012%) and ^41^K (6.730%). To date, although very few studies have done K isotope analysis in biological samples, K isotope ratios have shown much larger variations than geological samples (7–9). For instance, terrestrial plants show large K isotope differences between C_3_ (δ^41^K = −1.06 to -0.15‰) and C_4_ plants (δ^41^K = +0.28 to 1.15‰) (10). Moynier et al. (7) observed distinct pattern of K isotope ratios in animals with δ^41^K varying by ∼1‰ between the bovine blood and organs. Mahan et al. (8) studied the K isotope composition of brains from Göttingen minipigs and its implication by Alzheimer’s disease in humans. K isotope ratios for various minipig brain regions were found to range from −0.80 to −0.34‰, with a variability of ∼0.5‰ between different brain regions. Higgins et al. (11) described that the differences in K isotope fractionation is caused by the different classes of K^+^ transporter and their role in our body. It has been proposed that K^+^ transport via active channels and pores is associated with kinetic isotope fractionation, while transport via passive pumps and co-transporters causes negligible isotope effects (11). Until now only one study has surveyed K isotope composition associated to cancer, which showed similar δ^41^K for blood samples from cancer and control patients (12). However, there have been no reported studies investigating the isotopic composition of K in relation to endocrine pancreatic function and the development of diseases in humans.

In the human body, K^+^ ions are the second most abundant cation and one of the most important electrolytes. Dietary intake, excretion, intracellular and extracellular exchange maintain strict K^+^ homeostasis. While intracellular K^+^ dominates, only 2% of K^+^ can be found extracellularly (13). The distribution of K^+^ in the cells is regulated by K^+^ channels and pumps. K^+^ channels are classified in four main groups based on their functional and structural properties: Kv, calcium-activated K^+^ channels, inward-rectifier K^+^ channels and two-pore-domain K^+^ channels. These K^+^ channels control cell proliferation, hormone function and insulin excretion. Dysregulation of K^+^ channels can promote development and progression of many types of diseases, such as cardiovascular and Alzheimer’s diseases (14, 15), as well as various types of cancer (16). A recent study showed that electrolyte disturbances, including K^+^, can result in poorer overall survival rate of cancer patients (17). This demonstrates the importance of monitoring electrolyte disorders in various types of diseases.

K^+^ balance in the human body is controlled by K^+^ intake and K^+^ excretion about 90% of K^+^ is excreted through the urinary tract (18). About 90-95% of retained K^+^ is stored in muscle and bones (19, 20). In the pancreas, K^+^ regulates the insulin secretion. Potassium helps to regulate the electrical activity of beta cells, which is important for the synchronization of insulin secretion (21, 22). In addition, changes in K^+^ metabolism play an important role in the pancreatic pathology, such as in pancreatitis and pancreatic cancer. Difference in the expression of K-calcium-activated channel KCNN4 and over-expression of the voltage-activated potassium channel KCNH2 are associated with poorer prognosis of patients diagnosed with pancreatic cancer (21).

Pancreatic ductal adenocarcinoma (PDAC) is currently the fourth leading cause of cancer-related death but predicted to become the second by 2030 in the United States (23). The lack of a definitive markers to distinguish pancreatic cancer from other pancreatic diseases severely delays timely detection of patients with this malignancy.

We hypothesize that alteration of K^+^ transporting channels or pumps leads to dysregulated K^+^ homeostasis. In terms of K isotopes (23), Youn et al. (24) already suggested that urinary K isotope composition can help quantify *in vivo* K^+^ fluxes and trace mechanisms in physiological regulation of K^+^ homeostasis and also dyshomeostasis.

## 2 Methods

### 2.1 Sample collection and preparation for element analysis

For this study urine specimens were obtained from Barts Pancreas Tissue Bank, after patient consent and with ethical approval (reference number 18/NE/0070). Our pilot study includes 48 urine samples from three groups: patients with pancreatic cancer (PDAC), individuals with benign hepatobiliary diseases such as pancreatitis, cholelithiasis, cholecystitis (BE) and healthy controls (HC). Mid-stream urine was collected in 50 mL sterile containers.

Urine sample preparation was performed by microwave acid digestion. A 0.5 mL urine sample was transferred in acid cleaned 20mL MARSXpress vessels (PFA) vessels and 2 mL of concentrated ultra-trace nitric acid (HNO_3_ Optima grade, 65-67%wt, Fisher Scientific) was added. For quality control, blank samples consisting of only 2 mL of ultra-trace HNO_3_ were processed and two reference materials bovine muscle (ERM-BB184, 0.2 g dry weight) obtained from the European Commission and Seronorm blood (Seronorm™ Trace Elements in Whole Blood, 0.2 mL) were used because K isotope ratios have been reported previously (7, 9). The samples were digested using the MARS 6 Digestion Microwave System (CEM Corp., USA) by ramping up the temperature stepwise to 210°C, and held there for 30 minutes to ensure complete digestion. After cooling the digested samples were transferred to metal-free 15 mL Labcon PP centrifuge tubes and diluted to 10 mL with ultrapure water (18.2 MΩ*cm Hydro Picosystem).

### 2.2 Urinary potassium concentrations (sample preparation and analysis)

An Agilent 8900 Inductively Coupled Plasma triple quadrupole Mass Spectrometer (ICP-QQQ-MS) equipped with an Agilent SPS 4 autosampler system was used for total urinary K analysis. The standard ICPMS setup was employed for all experiments, consisting of a MicroMist quartz glass nebulizer, a glass double pass spray chamber, platinum/copper sampler and skimmer cones, and a quartz plasma torch with an inner diameter of 2.5 mm. The operating ICPMS parameters were as follows: radiofrequency (RF) power: 1,550 W; plasma gas: 15.0 L min^-1^; auxiliary gas: 0.9 L min^-1^; RF matching: 1.40; sampling depth: 8.0 mm; nebulizer gas flow rate: 0.95 L min^-1^; makeup gas (argon) flow rate: 0.20 L min^-1^; nebulizer pump speed: 0.10 rounds per second (~0.35 mL min^-1^); spray chamber temp.: 2°C. Potassium (m/z = 39) was measured in the ammonia mode (flow rate: 25%) and using the internal standard scandium (m/z = 45). Integration times for all masses were set to 0.1 seconds. External calibration was performed in matrix matched (aqueous 3% vol. HNO_3_, incl. 100 µg Sc/L) standards within the concentration range of from 5 to 5,000 µg K/L.

Urinary Zn concentrations were measured using PerkinElmer NexION 350S (Waltham, MA, USA) ICP-MS equipped with an Elemental Scientific (ESI) 4DX autosampler (Omaha, NE, USA). Zinc (m/z = 66) was measured with no collision gas and using the internal standard gallium (m/z = 69). Like K, external Zn calibration was performed in matrix matched standards ranging from 1 to 150 µg Zn/L.

The limit of detection (LOD) for K and Zn was calculated using the formula: LOD = 3.33 × standard deviation; the LOD was 2 µg K/L and 0.3 µg Zn/L measurement solution, respectively. Method detection limit (MDL) was calculated by multiplication of the LOD with the sample dilution factor. The MDL was 20 µg/L urine for K and 48 µg/L for Zn. Calculated accuracy and precision for total K were 98 ± 2%; and 95 ± 1% and 101 ± 2% for ^39^K and ^41^K, respectively; the certified value of Seronorm is 2034 ± 29 µg K^+^/L. For Zn the precision was 107 ± 10% (n=12) for QM-U-Q1823 (certified value = 281 µg Zn/L) and 101 ± 19% (n=11) for QM-U-Q1905 (certified value = 394 µg Zn/L); certified urine reference materials from the Quebec Multi-element External Quality Assessment Scheme (QMEQAS, Quebec, Canada).

To adjust for hydration status and dilution, urinary K^+^ and Zn concentrations were normalized to urine specific gravity (SG) using the Levin-Fahy (1945) equation: C_norm_ = C_measured_ (SG_ref_-1)/(SG_measured_-1) where C_measured_ is the measured urinary element concentrations and SG_measured_ is the sample urine specific gravity. SG_ref_ describes median value for healthy humans with reference urinary SG of 1.02 (25, 26).

### 2.3 Urinary potassium isotopes (sample preparation and analysis)

For the K isotope measurements, we used the the Sapphire multicollector inductively coupled plasma mass spectrometer (MC-ICPMS) by Nu Instruments with collision/reaction cell technology. In order to achieve high analytical precision, chromatography method was used to separate K+ from matrix in clean lab condition. First, approximately 1 mL digested urine sample was dried down and rehydrated in 0.7 N HNO_3_. The samples were then run through one cycle of “big column” followed by two cycles of “small column” ion-exchange chromatography. Both columns are filled with AG50-X8 100–200 mesh cation-exchange resin [see details by Chen et al (26)]. All ^40^Ar^+^ and Ar-hydride spectral interferences are effectively eliminated through a series of reactions with H_2_ and He gas in the collision/reaction cell. This allows the ^39^K^+^ and ^41^K^+^ ion beams to be measured directly at peak centers with high sensitivity (27). The K isotope values were expressed in the delta notation as δ^41/39^K (‰) relative to NIST SRM 3141a as reported in Equation 1:


(1) δK41(‰)=((K41/K39)sample(K41/K39)NIST−SRM3141a)×1000

The average δ^41^K data for reference materials bovine muscle ERM BB184 (-1.00 ± 0.04‰ 2sd, n = 6) and Seronorm whole blood (+0.89 ± 0.02‰, n = 3) are in good agreement with previously published values (7, 9). The analytical precision of δ^41^K is typically better than 0.02‰ (2 s.e.). The voltages of blank (2% HNO_3_) were typically ~1000 mV and ~80 mV for m/z = 39 and m/z = 41, respectively compared to the sample and standard voltages of 180,000 mV for m/z = 39 and 14,000 mV m/z = 41. The total procedure blanks through this study were measured by ICP-MS. The average procedural blank was 80 ng of K^+^, which accounts for less than 0.2% of total K in the sample

### 2.4 Statistical analysis

Statistical analysis including descriptive statistics and Mann–Whitney U test was performed using GraphPad Prism 9.3.1. The Receiver Operating Characteristics (ROC) curve analysis was performed in R version 3.5.1. The results are provided as mean (95% K^+^ peaks CI) and median values (IQR) and for categorical variables as percentages (%). *P*-values were considered statistically significant when < 0.05, and the degree of the differences are marked as: ^*^ *p* < 0.05, ^**^ *p* < 0.01, and ^***^ *p* < 0.001. Data outliers have been excluded from the statistical analysis using interquartile range.

## 3 Results and discussion

### 3.1 Potassium metabolism

**Figure 1:**
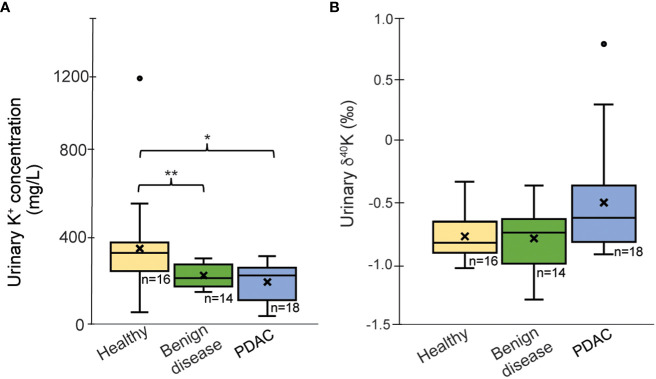
Boxplots of **(A)** K concentration (mg/L) corrected for urine-specify gravity and **(B)** δ^41/39^K_urine_ values for the healthy group, PDAC and patients with benign diseases. Outliers (°) have been excluded from the statistical calculations; “x’ reflects average values; ^*^p < 0.05, ^**^p < 0.01.

Urinary K^+^ concentrations are significantly lower in patients with benign diseases (236 ± 56 mg/L, *p=0.02*) and in PDAC patients (206 ± 56 mg/L; *p=0.004*) than in healthy controls (368 ± 259 mg/L) (**Figure 1A**).

Potassium isotopic data are summarized in **Table 1** and **Supplementary Table 2**. For urinary δ^41^K, the group of patients with benign pancreatic diseases exhibit an average value of -0.80 ± 0.27‰, which is very close to the values obtained for the healthy group. The urinary composition of PDAC patients ranges more widely, between +0.79‰ and -0.93‰ with a mean value of δ^41^K = -0.58 ± 0.33‰ (outlier excluded). Although PDAC patients tend to have more of ^41^K excreted in urine, the difference to the healthy controls did not reach statistical significance (*p=0.08*, **Figure 1B**).  A direct comparison between cancer stages is not possible due to the limited sample size.

**Table 1:** Number of participants, sex, age range, urinary K and Zn concentrations, and K isotopic composition, expressed as δ^41^K (‰) (IQR = interquartile range, 95% Cl = 95% confidence interval).

Variable	Healthy	Benign	PDAC
**Number of participants**	16	14	18
Diabetes	0	2	5
Women	38%	71%	50%
Men	62%	29%	50%
Age (years)			
Median (IQR)	50 (13)	48 (16)	65 (15)
Mean (95% CI)	50 (43, 57)	49 (43, 55)	64 (58, 70)
>50 years. (%)	57%	39%	94%
< 50 years (%)	43%	61%	6%
K concentrations (mg/L)			
Median (IQR)	349 (169)	222 (109)	237 (153)
Mean (95% CI)	368 (241, 495)	236 (207, 266)	203 (160, 246)
Zn concentrations (µg/L)			
Median (IQR)	602 (414)	492 (584)	947 (1250)
Mean (95% CI)	738 (498, 978)	607 (375, 839)	1207 (782, 1630)
δ^41^K (‰)			
Median (IQR)	-0.75 (0.23)	-0.84 (0.31)	-0.63 (0.43)
Mean (95% CI)	-0.78 (-0.87, -0.69)	-0.80 (-0.94, -0.66)	-0.58‰ (-0.72, -0.30)

Transport of K^+^ in and out of cells is carried out by K^+^ channels and K^+^ pumps. While channels transport K^+^ passively and thermodynamically downhill along the cell membrane, K^+^ transport via pumps is an active and thermodynamically uphill process. Higgins et al (11) proposed that these two groups of K^+^ cell transporters induce different K isotopic effects. While large isotope fractionation should be associated with K^+^ transport via channels, small isotope fractionation is expected for K^+^ transport through pumps (11).

In PDAC, it has been shown that several K^+^ channels are overexpressed, such as KCNN4, a K-Ca-activated channel (20, 21). Ion channels are size specific favoring the selection of ^39^K due to its smaller ionic radius and lower required transport energy compared to ^41^K. More specifically, ^41^K is 0.0035% larger than ^39^K ions (28) and this difference in ionic size can induce K isotopic fractionation of up to 1‰ via a selective transport. Alteration of K^+^ channels (e.g., dysfunction, overexpression) could explain the lower urinary excretion of K^+^ and more ^41^K for PDAC patients even though the difference in urinary K isotopes is only marginally significant compared to healthy controls. This would result in higher K^+^ levels and isotopically light K^+^ composition in pancreatic cells. Similar effects have been observed for Ca^2+^ isotope in plants where ion channels preferentially select isotopically light Ca^2+^ and induce an isotope fractionation of 1–2‰ (28).

Kidneys regulate and maintain K^+^ homeostasis via K excretion and re-adsorption and one might argue that any change in kidney function in diseased kidneys can cause the observed change in urinary K^+^ levels and K isotope composition. One fifth of pancreatic cancer patients also suffer from chronic kidney disease (28) and urinary K^+^ excretion might be diminished due to higher K^+^ re-adsorption. We believe that the urinary K isotope signal is cancer-mediated because glomerular filtration is not expected to fractionate K isotopes (11) and re-absorption preferentially retains ^41^K in the body. The trend in higher δ^41^K for PDAC compared to healthy controls does not follow the expected K isotopic shift.

### 3.2 Diabetes

**Figure 2:**
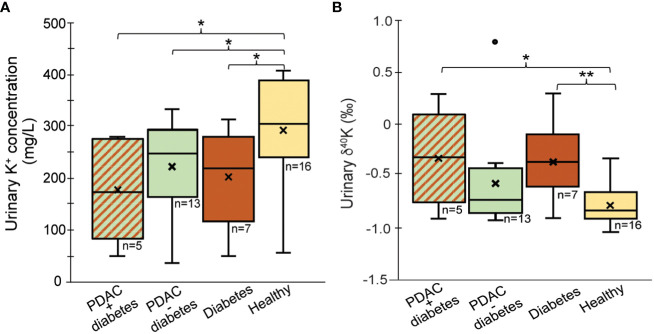
Comparison of **(A)** urine K^+^ concentration and **(B)** δ^41^K_urine_ values for patients with PDAC + diabetes, PDAC without diabetes and diabetes only. Outliers (°) have been excluded from the statistical calculations; “x’ reflects average values; *p < 0.05, **p < 0.01.

Because of known association of PDAC and diabetes, we also evaluated the relationship of type 2 diabetes with K^+^ levels and δ^41^K in urine. The diabetes group included two patients with benign pancreatic disease and five PDAC patients. Our data show that urinary K^+^ levels are lower in patients with diabetes (203 ± 95 mg/L) compared to healthy controls 368 ± 259 mg/L, (*p*=0.03; **Figure 2A**). Although the sample size is very small, urinary K^+^ levels for patients with PDAC and no diabetes are similar to the group of patients with diabetes regardless of PDAC status (*p = 0.53*). Diabetics excrete higher levels of ^41^K relative to healthy controls (*p*=0.009; **Figure 2B**). Although δ^41^K for patients with PDAC and no diabetes are not significantly different (*p=0.08*), there seems to be a trend that PDAC patients with diabetes excrete slightly higher levels of ^41^K (δ^41^K = -0.37 ± 0.39‰). Insulin is an important factor controlling the K^+^ transport across the cell under normal condition. As most cells, pancreatic cells have Na-K-ATPase pumps, which actively pump Na out of the cell and K^+^ into the cell. These ATP-sensitive K^+^ pumps regulate insulin secretion in pancreatic cells. Diabetes leads to an increased glucose metabolism which in response causes ATP increase and the dysregulation of these K^+^ pumps (29). As K isotope fractionation is expected to be small for K^+^ transport through pumps, the urinary shift in K isotopic ratios might be small and most likely overprinted by the high K^+^ levels in urine. That means even though diabetes leads to K pumps dysregulation, this changing effect is neither reflected in urinary K^+^ level nor in K isotope ratio. We would like to confirm this observation with a larger cohort.

### 3.3 Correlation of δ^41^K with zinc

**Figure 3:**
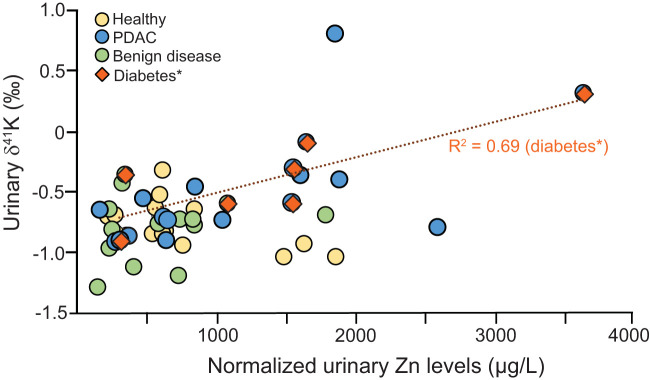
Correlation between Zn and δ^41^K for healthy controls (yellow), PDAC (blue), benign diseases (green)] and diabetes (brown) *diabetes includes urine from PDAC and benign disease group.

**Figure 3** shows the relationship of δ^41^K and Zn showing that low urinary zinc is associated with lower δ^41^K values. Zinc is essential for insulin synthesis and secretion, while K^+^ plays a role in the regulation of insulin secretion as well as the exocrine function of the pancreas. Imbalances in these elements can lead to impaired pancreatic function and a range of health problems, including diabetes. We have previously reported changes in urinary Zn in patients diagnosed with PDAC (4).

This correlation is particularly strong for patients diagnosed with diabetes. Both Zn and K^+^ are tightly regulated but reduced pancreatic Zn concentrations have been associated with type 2 diabetes (30) while Zn supplementation has insulin-enhancing effects and improves β-cell function (31) controlled by Na-K-ATPase pumps. Being able to measure the association between Zn and K^+^ in urine of diabetics can help unravel further metabolic dysregulation and potentially improve diabetes treatments.

### 3.4 Biomarker combination in differentiating control groups from PDAC

Finally, we wanted to check if combining the K^+^ results with our previous metal element data (4) would improve our three-biomarker panel for earlier detection of PDAC (32) and the associated algorithm for data interpretation PancRISK score (33).

**Figure 4:**
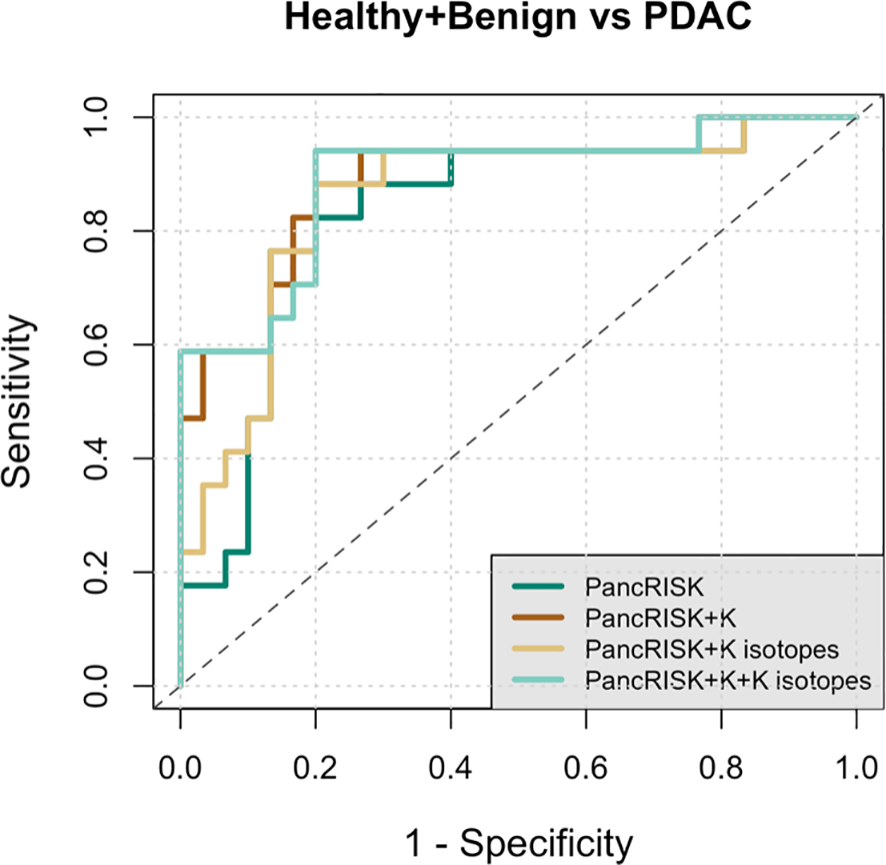
Receiver Operating Characteristic (ROC) curve analysis of data from healthy + benign and pancreatic cancer urine samples. Performance characteristics for PancRISK (dark green), PancRISK + K concentrations (brown), PancRISK + K isotopes (yellow) and PancRISK + K concentration + K isotopes (light green). The area under the ROC curve (AUC), sensitivity and specificity and the 95% confidence interval are listed in the table.

The Receiver Operating Characteristics (ROC) curves below (**Figure 4**) show true positive *vs.* false positive results for δ^41^K and/or a combination of urinary K^+^ levels and δ^41^K with our previously reported urinary biomarkers. The area under the ROC curve (AUC) indicates good accuracy in differentiating between controls and cancer patients when previous panel of three urinary proteins (PancRISK) is combined with both urinary K^+^ levels and K isotopic values (AUC = 0.89, 95% CI 0.79-0.99).

We acknowledge that our study has some limitations. The first is that the sample size is small (N=48) especially for patients with diabetes (N=7). However, this is the first report on δ^41^K for human urine and for a disease case-control cohort. Thus, interpretations drawn from the K isotope results should be considered exploratory and hypothesis-generating for future studies. Second, metabolic processes causing K isotope fractionation at different parts of the body at healthy and diseases states are understudied. Therefore, we can only provide a descriptive analysis of urinary δ^41^K associated with healthy or diseased pancreas. Finally, we did not have associated dietary information and differences in diet which could potentially affect urinary K isotope compositions.

## 4 Conclusion

Our pilot results were the first of such kind, where we have explored K isotopic composition in urine of patients with pancreatic diseases and compared them to healthy controls. This study also makes one of the first steps towards quantifying K isotope ratios in human systems, as the K isotope compositions of human blood and tissues are not well constrained. Additionally, as kidneys are the major path in K^+^ homeostasis in-depth biological studies are needed in order to understand effect of kidney function on K isotope fractionation (e.g., chronic kidney diseases). While additional K^+^ isotopic measurements in larger number of urine specimens are needed to validate the obtained results, K isotopes in urine might be a potentially powerful marker for an individualized risk stratification and detection of patients with diabetes and pancreatic cancer as other pancreatic diseases seem not to alter the urinary K^+^ levels and δ^41^K.

## Data availability statement

The original contributions presented in the study are included in the article/**Supplementary Material**. Further inquiries can be directed to the corresponding author.

## Ethics statement

The studies involving humans were approved by North East - York Research Ethics Committee; REC reference 18/NE/0070. The studies were conducted in accordance with the local legislation and institutional requirements. The human samples used in this study were acquired from gifted from another research group. Written informed consent for participation was not required from the participants or the participants’ legal guardians/next of kin in accordance with the national legislation and institutional requirements.

## Author contributions

KS: Formal analysis, Methodology, Writing – original draft. HC: Methodology, Writing – review & editing. RG: Methodology, Writing – review & editing. SD: Writing – review & editing. OB: Visualization, Writing – review & editing. AN-A: Writing – review & editing. AH: Writing – review & editing. TC-J: Writing – review & editing, Resources.

## Supplementary material

Supplementary Table 2 Pancreatic cancer, other pancreatic diseases (pancreatitis and benign cancer) and healthy controls: age, sex (F=female and M=male), diabetes (yes=y and no=n), urine specific gravity (SG), SG-normalized urinary K and Zn concentrations and K isotopes values d41K. - = no data available.

## References

[1] Larner F Woodley LN Shousha S Moyes A Humphreys-Williams E Strekopytov S. Zinc isotopic compositions of breast cancer tissue. Metallomics. (2015) 7:112–7. doi: 10.1039/C4MT00260A 25489714

[2] Toubhans B Gourlan AT Telouk P Lutchman-Singh K Francis LW Conlan RS. Cu isotope ratios are meaningful in ovarian cancer diagnosis. J Trace Elem Med Bio. (2020) 62. doi: 10.1016/j.jtemb.2020.126611 32652467

[3] Schilling K Moore RET Sullivan KV Capper MS Rehkämper M Goddard K. Zinc stable isotopes in urine as diagnostic for cancer of secretory organs. Metallomics. (2021) 13. doi: 10.1093/mtomcs/mfab020 33877364

[4] Schilling K Larner F Saad A Roberts R Kocher HM Blyuss O. Urine metallomics signature as an indicator of pancreatic cancer. Metallomics. (2020) 12:752–7. doi: 10.1039/d0mt00061b 32211672

[5] Sullivan KV Moore RET Capper MS Schilling K Goddard K Ion C. Zinc stable isotope analysis reveals Zn dyshomeostasis in benign tumours, breast cancer, and adjacent histologically normal tissue. Metallomics. (2021) 13. doi: 10.1093/mtomcs/mfab027 33970272

[6] Tani LSK Gourlan AT Dennouni-Medjati N Telouk P Dali-Sahi M Harek Y. Copper isotopes and copper to zinc ratio as possible biomarkers for thyroid cancer. Front Med-Lausanne. (2021) 8. doi: 10.3389/fmed.2021.698167 PMC8455850 34568365

[7] Moynier F Hu Y Dai W Kubik E Mahan B Moureau J. Potassium isotopic composition of seven widely available biological standards using collision cell (CC)-MC-ICP-MS. J Anal Atom Spectrom. (2021) 36:2444–8. doi: 10.1039/D1JA00294E

[8] Mahan B Tacail T Lewis J Elliott T Habekost M Turner S. Exploring the K isotope composition of Gottingen minipig brain regions, and implications for Alzheimer's disease. Metallomics. (2022) 14. doi: 10.1093/mtomcs/mfac090 PMC9764214 36416864

[9] Hobin K Rodriguez MC Vanhaecke F. Robust potassium isotopic analysis of geological and biological samples via multicollector ICP-mass spectrometry using the "Extra-high resolution mode". Anal Chem. (2021) 93:8881–8. doi: 10.1021/acs.analchem.1c01087 34133117

[10] Qu R. HG. Potassium isotopes in herbaceous plants: A potential new tool for C3 and C4 plant research. JGR Biogeosciences. (2022) 127:e2021JG006682. doi: 10.1029/2021JG006682

[11] Higgins JA Ramos DS Gili S Spetea C Kanoski S Ha D. Stable potassium isotopes ((41)K/(39)K) track transcellular and paracellular potassium transport in biological systems. Front Physiol. (2022) 13:1016242. doi: 10.3389/fphys.2022.1016242 36388124 PMC9644202

[12] Télouk P Albalat E Tacail T Arnaud-Godet F Balter V. Steady analyses of potassium stable isotopes using Thermo Scientific Neoma MC-ICP-MS. J Anal At Spectrom. (2022) 37:1259–64. doi: 10.1039/D2JA00050D

[13] Youn JH McDonough AA. Recent advances in understanding integrative control of potassium homeostasis. Annu Rev Physiol. (2009) 71:381–401. doi: 10.1146/annurev.physiol.010908.163241 18759636 PMC4946439

[14] Sobey CG. Potassium channel function in vascular disease. Arterioscl Throm Vas. (2001) 21:28–38. doi: 10.1161/01.ATV.21.1.28 11145930

[15] de Silva HA Aronson JK Grahame-Smith DG Jobst KA Smith AD. Abnormal function of potassium channels in platelets of patients with Alzheimer's disease. Lancet. (1998) 352:1590–3. doi: 10.1016/S0140-6736(98)03200-0 9843105

[16] Cantini L Merloni F Rinaldi S Lenci E Marcantognini G Meletani T. Electrolyte disorders in advanced non-small cell lung cancer patients treated with immune check-point inhibitors: A systematic review and meta-analysis. Crit Rev Oncol Hematol. (2020) 151:102974. doi: 10.1016/j.critrevonc.2020.102974 32416348

[17] Ingles Garces AH Ang JE Ameratunga M Chénard-Poirier M Dolling D Diamantis N. A study of 1088 consecutive cases of electrolyte abnormalities in oncology phase I trials. Eur J Cancer. (2018) 104:32–8. doi: 10.1016/j.ejca.2018.08.019 PMC6259582 30316017

[18] Udensi UK Tchounwou PB. Potassium homeostasis, oxidative stress, and human disease. Int J Clin Exp Physiol. (2017) 4:111–22. doi: 10.4103/ijcep.ijcep_43_17 PMC5716641 29218312

[19] McLean RM Wang NX. Potassium. Adv Food Nutr Res. (2021) 96:89–121. doi: 10.1016/bs.afnr.2021.02.013 34112360

[20] Leggett RW Williams LR. A model for the kinetics of potassium in healthy humans. Phys Med Biol. (1986) 31:23–42. doi: 10.1088/0031-9155/31/1/003 3952144

[21] Jiang SH Zhu LL Yang JY Hu L Gu J Xing X. Integrated expression profiling of potassium channels identifys KCNN4 as a prognostic biomarker of pancreatic cancer. Biochem Bioph Res Co. (2017) 494:113–9. doi: 10.1016/j.bbrc.2017.10.072 29050937

[22] Hayashi M Novak I. Molecular basis of potassium channels in pancreatic duct epithelial cells. Channels (Austin). (2013) 7:432–41. doi: 10.4161/chan.26100 PMC4042478 23962792

[23] Rahib L Smith BD Aizenberg R Rosenzweig AB Fleshman JM Matrisian LM. Projecting cancer incidence and deaths to 2030: the unexpected burden of thyroid, liver, and pancreas cancers in the United States. Cancer Res. (2014) 74:4006–6. doi: 10.1158/0008-5472.CAN-14-1642 24840647

[24] Youn JH Oh YT Gili S McDonough AA Higgins J. Estimating *in vivo* potassium distribution and fluxes with stable potassium isotopes. Am J Physiol-Cell Ph. (2022) 322:C410–20. doi: 10.1152/ajpcell.00351.2021 PMC8917925 35080924

[25] Burton C Dan Y Donovan A Liu K Shi H Ma Y. Urinary metallomics as a novel biomarker discovery platform: Breast cancer as a case study. Clin Chim Acta. (2016) 452:142–8. doi: 10.1016/j.cca.2015.11.014 26585752

[26] ETM R Rehkamper M Kreissig K Strekopytov S Larner F. Determination of major and trace element variability in healthy human urine by ICP-QMS and specific gravity normalisation. RSC Adv. (2018) 8:38022–35. doi: 10.1039/C8RA06794E PMC9089848 35558613

[27] Chen H Saunders NJ Jerram M Halliday AN. High-precision potassium isotopic measurements by collision cell equipped MC-ICPMS. Chem Geol. (2021) 578. doi: 10.1016/j.chemgeo.2021.120281

[28] Christensen JN Qin LP Brown ST DePaolo DJ. Potassium and Calcium Isotopic Fractionation by Plants (Soybean [Glycine max], Rice [Oryza sativa], and Wheat [Triticum aestivum]). ACS Earth Space Chem. (2018) 2:745–52. doi: 10.1021/acsearthspacechem.8b00035

[29] Ashcroft FM Rorsman P. K(ATP) channels and islet hormone secretion: new insights and controversies. Nat Rev Endocrinol. (2013) 9:660–9. doi: 10.1038/nrendo.2013.166 PMC5890885 24042324

[30] Chausmer AB. Zinc, insulin and diabetes. J Am Coll Nutr. (1998) 17:109–15. doi: 10.1080/07315724.1998.10718735 9550453

[31] Maret W. Zinc in pancreatic islet biology, insulin sensitivity, and diabetes. Prev Nutr Food Sci. (2017) 22:1–8. doi: 10.3746/pnf.2017.22.1.1 28401081 PMC5383135

[32] Debernardi S O'Brien H Algahmdi AS Malats N Stewart GD Plješa-Ercegovac M. A combination of urinary biomarker panel and PancRISK score for earlier detection of pancreatic cancer: A case-control study. PloS Med. (2020) 17:e1003489. doi: 10.1371/journal.pmed.1003489 33301466 PMC7758047

[33] Blyuss O Zaikin A Cherepanova V Munblit D Kiseleva EM Prytomanova OM. Development of PancRISK, a urine biomarker-based risk score for stratified screening of pancreatic cancer patients. Br J Cancer. (2020) 122:692–6. doi: 10.1038/s41416-019-0694-0 PMC7054390 31857725

