# Supplementary figures and images for: Analysis of urinary potassium isotopes and association with pancreatic health: healthy, diabetic and cancerous states
Source: Front Endocrinol (Lausanne). 2024 Apr 2;15:1332895. doi: 10.3389/fendo.2024.1332895 (PMC11062322; doi:10.3389/fendo.2024.1332895)

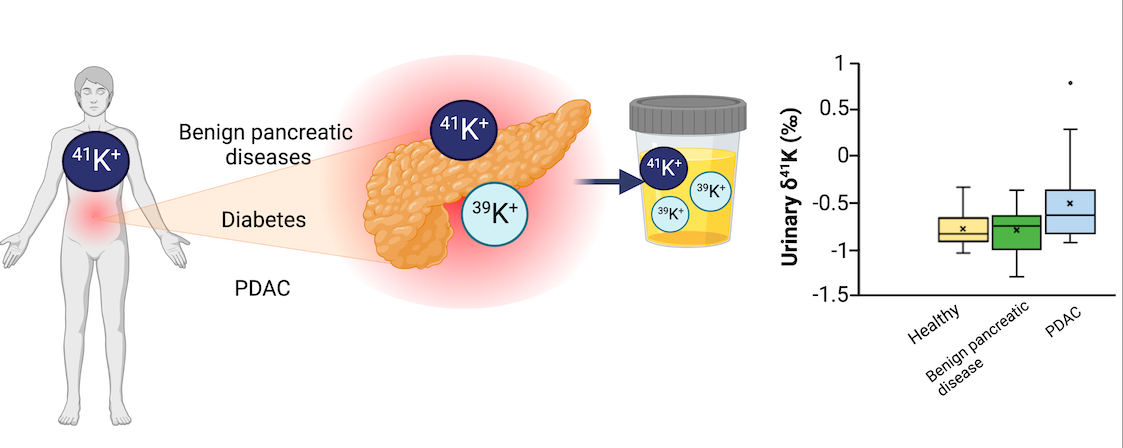

Supplement: Supplementary file 3 [file Image_1.png]
